# Supplementary material for: PKPy: a Python-based framework for automated population pharmacokinetic analysis
Source: PeerJ. 2025 Oct 27;13:e20258. doi: 10.7717/peerj.20258 (PMC12574595; doi:10.7717/peerj.20258)
Supplement: Supplemental Information 1 — Parameter estimates (Clearance, Volume of distribution, and Absorption rate constant) obtained using PKPy and Saemix software for the Theophylline dataset (12 subjects receiving a single oral dose of 320 mg). PKPy results are shown both with and without initial parameter estimates to demonstrate the effectiveness of automated initialization. [file peerj-13-20258-s001.docx]

Supplement 1.

< No initial parameters provided >

Column Classifications:

--------------------------------------------------

ID Column: Subject

Time Column: Time

Concentration Column: conc

Covariate Columns:

- Wt

- Dose

NCA analysis completed. Success rate: 12/12 subjects

Metrics calculated using 132 valid data points

===============================================

PK Analysis Summary

===============================================

FINAL PARAMETER ESTIMATES

-----------------------------------------------

Parameter Estimate CV% [95% CI]

-----------------------------------------------

Ka 1.284 121.8 [0.555, 8.179]

CL 2.794 23.3 [1.627, 3.869]

V 31.732 17.8 [23.648, 41.263]

BETWEEN-SUBJECT VARIABILITY (CV%)

-----------------------------------------------

Ka 79.8%

CL 26.3%

V 18.0%

RESIDUAL VARIABILITY

-----------------------------------------------

Proportional Error (CV%): 14.4%

GOODNESS-OF-FIT STATISTICS

-----------------------------------------------

R-squared: 0.933

RMSE: 0.740

MAE: 0.428

Mean Residual: 0.098

NCA PARAMETERS SUMMARY

-----------------------------------------------

Cmax Tmax AUC AUMC MRT half_life clearance \

count 12.000 12.000 12.000 12.000 12.000 12.000 12.000

mean 8.759 1.788 103.807 883.057 8.410 8.149 3.222

std 1.473 1.112 23.645 262.982 0.594 2.119 0.679

min 6.440 0.630 73.776 609.152 7.707 6.287 2.149

25% 7.890 1.015 88.002 706.247 8.135 6.927 2.660

50% 8.465 1.135 95.407 792.803 8.304 7.670 3.360

75% 9.865 2.385 120.307 987.689 8.483 8.409 3.637

max 11.400 3.550 148.923 1459.071 9.797 14.304 4.337

Vss Subject_ID Ka CL V

count 12.000 12.000 12.000 12.000 12.000

mean 26.840 5.500 2.054 2.884 32.346

std 4.735 3.606 2.613 0.702 6.002

min 21.052 0.000 0.541 1.528 22.872

25% 22.027 2.750 0.799 2.512 28.597

50% 26.532 5.500 1.143 3.057 31.792

75% 30.312 8.250 1.666 3.248 37.819

max 35.814 11.000 9.842 3.948 41.773

NCA Success Rate: 100.0%

SIGNIFICANT COVARIATES

-----------------------------------------------

Ka-Dose: power

V-Wt: power


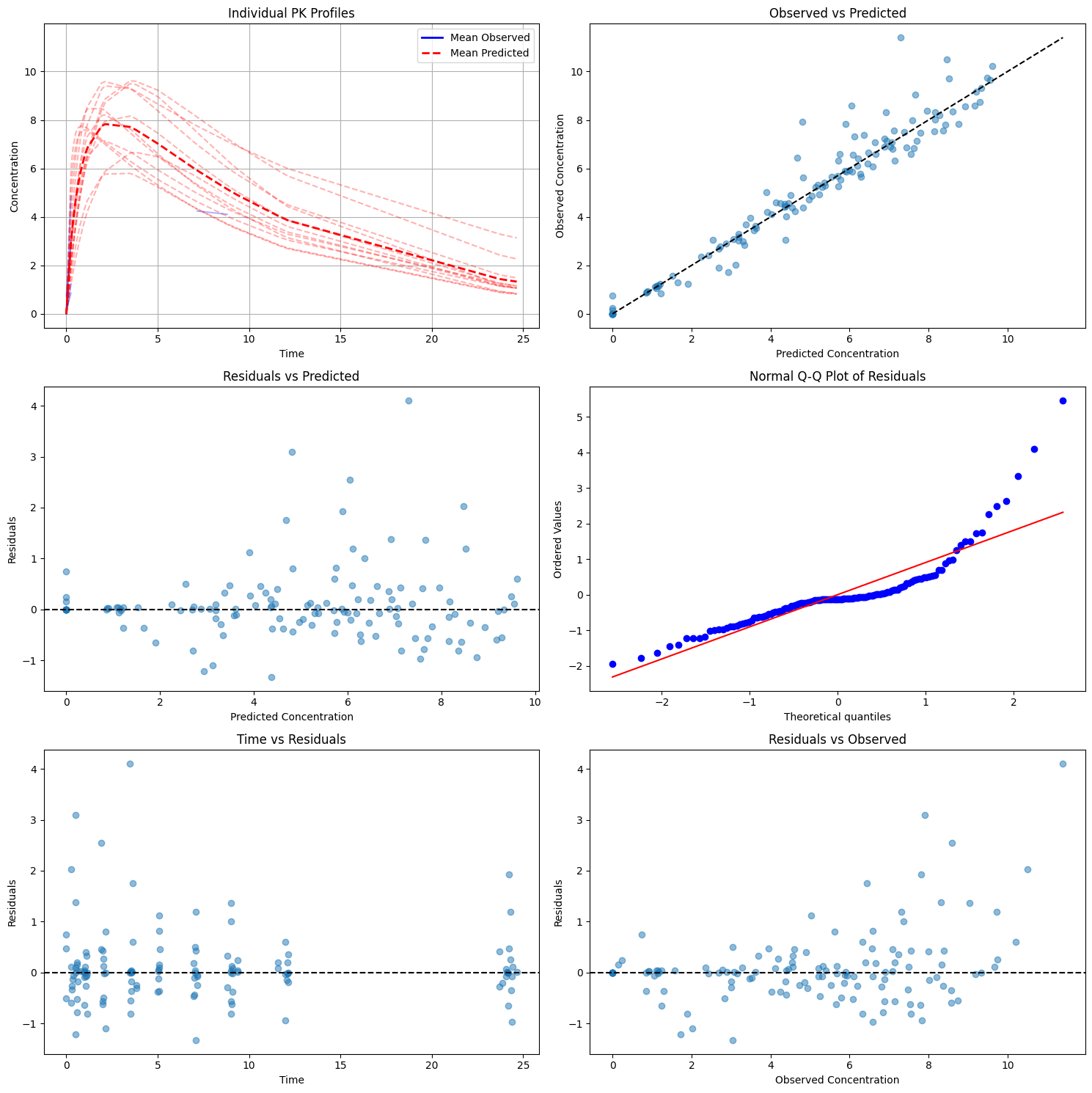


< Provide initial parameters >

Column Classifications:

--------------------------------------------------

ID Column: Subject

Time Column: Time

Concentration Column: conc

Covariate Columns:

- Wt

- Dose

NCA analysis completed. Success rate: 12/12 subjects

Metrics calculated using 132 valid data points

===============================================

PK Analysis Summary

===============================================

FINAL PARAMETER ESTIMATES

-----------------------------------------------

Parameter Estimate CV% [95% CI]

-----------------------------------------------

Ka 1.284 80.5 [0.555, 4.668]

CL 2.794 23.3 [1.627, 3.869]

V 31.732 17.4 [23.648, 40.865]

BETWEEN-SUBJECT VARIABILITY (CV%)

-----------------------------------------------

Ka 67.0%

CL 26.3%

V 17.7%

RESIDUAL VARIABILITY

-----------------------------------------------

Proportional Error (CV%): 14.4%

GOODNESS-OF-FIT STATISTICS

-----------------------------------------------

R-squared: 0.933

RMSE: 0.740

MAE: 0.428

Mean Residual: 0.098

NCA PARAMETERS SUMMARY

-----------------------------------------------

Cmax Tmax AUC AUMC MRT half_life clearance \

count 12.000 12.000 12.000 12.000 12.000 12.000 12.000

mean 8.759 1.788 103.807 883.057 8.410 8.149 3.222

std 1.473 1.112 23.645 262.982 0.594 2.119 0.679

min 6.440 0.630 73.776 609.152 7.707 6.287 2.149

25% 7.890 1.015 88.002 706.247 8.135 6.927 2.660

50% 8.465 1.135 95.407 792.803 8.304 7.670 3.360

75% 9.865 2.385 120.307 987.689 8.483 8.409 3.637

max 11.400 3.550 148.923 1459.071 9.797 14.304 4.337

Vss Subject_ID Ka CL V

count 12.000 12.000 12.000 12.000 12.000

mean 26.840 5.500 1.650 2.884 32.225

std 4.735 3.606 1.387 0.702 5.848

min 21.052 0.000 0.541 1.528 22.873

25% 22.027 2.750 0.799 2.512 28.597

50% 26.532 5.500 1.143 3.057 31.791

75% 30.312 8.250 1.666 3.250 37.819

max 35.814 11.000 5.000 3.948 41.774

NCA Success Rate: 100.0%

SIGNIFICANT COVARIATES

-----------------------------------------------

Ka-Dose: power

V-Wt: power

===============================================

============================================

NCA Analysis Summary

============================================

Analysis Success Rate: 100.0%

Parameter Estimates

--------------------------------------------

Cmax:

N = 12 (missing: 0)

Arithmetic mean ± SD: 8.759 ± 1.410

CV%: 16.1

Geometric mean (CV%): 8.646 (16.2%)

Median: 8.465

Range: [6.440, 11.400]

Tmax:

N = 12 (missing: 0)

Arithmetic mean ± SD: 1.788 ± 1.065

CV%: 59.6

Geometric mean (CV%): 1.515 (61.4%)

Median: 1.135

Range: [0.630, 3.550]

AUC:

N = 12 (missing: 0)

Arithmetic mean ± SD: 103.807 ± 22.639

CV%: 21.8

Geometric mean (CV%): 101.482 (21.3%)

Median: 95.407

Range: [73.776, 148.923]

half_life:

N = 12 (missing: 0)

Arithmetic mean ± SD: 8.149 ± 2.029

CV%: 24.9

Geometric mean (CV%): 7.955 (20.9%)

Median: 7.670

Range: [6.287, 14.304]

clearance:

N = 12 (missing: 0)

Arithmetic mean ± SD: 3.222 ± 0.650

CV%: 20.2

Geometric mean (CV%): 3.153 (21.3%)

Median: 3.360

Range: [2.149, 4.337]

MRT:

N = 12 (missing: 0)

Arithmetic mean ± SD: 8.410 ± 0.569

CV%: 6.8

Geometric mean (CV%): 8.392 (6.6%)

Median: 8.304

Range: [7.707, 9.797]

Vss:

N = 12 (missing: 0)

Arithmetic mean ± SD: 26.840 ± 4.533

CV%: 16.9

Geometric mean (CV%): 26.462 (17.0%)

Median: 26.532

Range: [21.052, 35.814]

Quality Checks

--------------------------------------------

AUC extrapolation:

Mean %: nan

Subjects with >20% extrapolation: 0

Sampling Schedule:

Minimum time gap (absorption): 0.02 h

Maximum time gap (elimination): 0.22 h

Total observation period: 24.6 h
